# Exploring Readiness for Birth Control in Improving Women Health Status: Factors Influencing the Adoption of Modern Contraceptives Methods for Family Planning Practices

**DOI:** 10.3390/ijerph182211892

**Published:** 2021-11-12

**Authors:** Adnan Muhammad Shah, KangYoon Lee, Javaria Nisa Mir

**Affiliations:** 1 Department of Computing Engineering, Gachon University, Seoul 13120, Korea; adnan.shah@gachon.ac.kr; 2 Department of Management Sciences, Shaheed Zulfikar Ali Bhutto Institute of Science and Technology, Islamabad 44320, Pakistan; 3 Charles E. Schmidt College of Science, Florida Atlantic University, Boca Raton, FL 33431, USA; 4 Faculty of Management Science, Riphah International University, Rawalpindi 46000, Pakistan; javeriamir011@gmail.com

**Keywords:** modern contraceptives, perceived barriers, sexual and reproductive health, birth control methods

## Abstract

Background: Pakistan is the world’s sixth most populated country, with a population of approximately 208 million people. Despite this, just 25% of legitimate couples say they have used modern contraceptive methods. A large body of literature has indicated that sexual satisfaction is a complex and multifaceted concept, since it involves physical and cultural components. The purpose of this study is to investigate the impact of influencing factors in terms of contraceptive self-efficacy (CSE), contraceptive knowledge, and spousal communication on the adoption of modern contraceptive methods for family planning (FP) under the moderating role of perceived barriers. Methods: Data were collected using an adopted questionnaire issued to married women of reproductive age belonging to the Rawalpindi and Neelum Valley regions in Pakistan. The sample consisted of 250 married women of reproductive age. SPSS was used to analyze the respondents’ feedback. Results: The findings draw public attention towards CSE, contraceptive knowledge, and spousal communication, because these factors can increase the usage of modern methods for FP among couples, leading to a reduction in unwanted pregnancies and associated risks. Regarding the significant moderation effect of perceived barriers, if individuals (women) are highly motivated (CSE) to overcome perceived barriers by convincing their husbands to use contraceptives, the probability to adopt modern contraceptive methods for FP practices is increased. Conclusions: Policymakers should formulate strategies for the involvement of males by designing male-oriented FP program interventions and incorporating male FP workers to reduce communication barriers between couples. Future research should address several other important variables, such as the desire for additional child, myths/misconceptions, fear of side effects, and partner/friend discouragement, which also affect the adoption of modern contraceptive methods for FP practices.

## 1. Background

Pakistan is the world’s sixth most populated country, with a population of 208 million people at the time of writing [1]. The Pakistani government is concerned about population growth because it is related to economic and social consequences of unrestrained expansion [2,3]. Failure to control the rate of reproduction and rapid population expansion has negative consequences for development indices such as education, poverty, and life expectancy, especially for mother and child health [4]. Beginning in the 1960s, the country became a pioneer in the field of family planning (FP) among developing countries. Fifty years later, the program is still struggling to increase the use of modern contraceptives. The current contraceptive prevalence rate in Pakistan is 34%, compared to 62% in India and 56% in Bangladesh [5,6]. For years, the low and stagnant prevalence of contraception in Pakistan has been a source of academic debate [7]. Much has been written about Pakistan’s sluggish adoption of modern contraception methods, highlighting cultural hurdles, inconsistent political support, and service delivery failures [7,8]. The majority of the research has focused on service delivery problems, with the assumption that increasing contraceptive provision would improve contraceptive use [8,9,10,11].

The gradual increase in contraceptive rates in Pakistan compared to other nations in the region has been a hotly debated topic among demographers and other academics, with many speculating that inconsistencies in political support and a lackluster FP policy are to blame [11,12]. Researchers recommend that communication between couples should be encouraged because it increases the adoption of FP practices [13,14,15]. A recent study indicated that there is a need for modern contraceptive prevalence in Pakistan, which requires an increased uptake of contraceptives (National Institute of Population Studies (NIPS)) [16]. Pakistan has been facing the issue of FP for decades [17]. About 17% of married women in Pakistan have modern contraceptive prevalence for FP, and this rate is higher among rural areas. The demand for FP has reduced over the last 5 years, currently at 52% whilst it was 55% in 2012–2013. Pakistan has a 34% contraceptive prevalence rate, and the use of modern contraceptive methods has not increased since 2013 [16]. The literature shows that knowledge on contraceptives has profound effects on the FP practices [18]. Due to a lack of appropriate knowledge about contraceptive methods, women cannot get desired results [19].

Women’s self-efficacy and knowledge about the appropriate use and the side effects of contraceptive methods, a couple’s communication, and combined decisions are positive predictors of contraceptive use [20]. Women’s education and power to make decisions are significantly associated with the use of contraceptives [21]. Previous literature has indicated low contraceptive use in Pakistan, and there is an urgent need to explore factors which can help to improve FP practices and modern contraceptive prevalence necessary for FP practices [22]. Contraceptive self-efficacy (CSE), contraceptive knowledge, and spousal communication are found to be associated with FP practices [23]. Self-efficacy theory suggests that an individual’s belief in his own competence to perfectly perform any behavior is affected by several moderators and barriers, either personal or social [24]. Therefore, researchers have suggested that while assessing self-efficacy, the impact of perceived barriers on health behavior estimation must be examined [25]. Researchers have also reported several reasons for why improving contraceptive knowledge might improve contraceptive use [26]. Spousal communication is the determinant of FP practices, but there is need to assess this connection in the context of developing countries [13]. Because a lack of communication and counselling is affecting couples’ and women’s decision-making ability regarding fertility preferences [14], the current study attempts to assess the impact of these variables on women’s perceptions regarding the adoption of modern contraceptive methods for FP practices.

Numerous economists and researchers continue to doubt Pakistan’s ability to significantly boost the adoption of modern FP practices because of religious norms, social liberalism, and preferences for large family systems. Therefore, several gaps are observed in the policies and structure of programs related to FP practices in Pakistan [8,11] and other developing regions [27,28]. The unavailability of contraceptives, especially in rural areas, users’ dissatisfaction, low service quality, lack of proper guidance concerning the methods selected, religious factors, and a lack of knowledge, funding, and collaboration between public and private sector facilities providing FP services have been quoted as barriers that cause a low prevalence of contraceptive measures [10,17]. Since the context of this study is Pakistan, it is worth noting that FP in Pakistan is entirely female-oriented [29]. Programs that target only a single sex tend to fail to achieve its targets [13]. Therefore, all these issues need to be investigated, because they are affecting population control activities in the country. The theoretical foundation of this study is based on a combined health belief model, social cognitive theory, and the theory of planned behavior. In this regard, this study attempts to examine different predictors in the adoption of modern contraceptive methods for FP practices. This study will provide a thorough understanding of these factors, which will be helpful for the control of fertility.

The current study aims to explore the impact of spousal communication, contraceptive knowledge, and CSE on the adoption of modern contraceptive methods for FP practices in a developing country context, such as Pakistan. In addition, the moderating role of perceived barriers is, for the first time, theorized and tested to determine the relationship between contraceptive knowledge, spousal communication, CSE, and the adoption of modern contraceptive methods for FP practices. The findings of the current study would be helpful for policymakers in implementing and revising policies to further improve FP programs.

The rest of the sections in the current study are arranged as follows: Section 2 presents a literature review and hypotheses; Section 3 covers the proposed methodology, including sample and data collection, the measurement of variables, common method bias, and control variables; Section 4 explains the data analysis and results; finally, Section 5 discusses the results of the study, sheds light on practical implications, and recommends a direction for future research.

## 2. Literature Review

### 2.1. Contraceptive Self-Efficacy (CSE) and Family Planning (FP) Practices

Levinson, as cited in [30], defined CSE as “it is the strength of a young woman’s conviction that she should and could exercise control within sexual and contraceptive situations to prevent an unintended pregnancy, if that is what she desires” (p. 9). Following the self-efficacy theory, the concept of CSE was developed to measure women’s self-efficacy and its impact on their reproductive health. The extant literature indicates that women with higher self-efficacy are more independent in the selection and practice of modern contraceptive methods [31,32]. CSE is important because it stimulates individual behavior related to the use of modern contraceptives, therefore helping to prevent major public health issues by prompting the use of modern contraceptives [31]. Contraceptive acceptance is higher among females with higher CSE [33,34,35]. CSE enables women to manage all resistance related to FP practices [25]. Findings from previous research also reveal that CSE increases contraceptive adherence [20]. The above explanations suggest that CSE is a strong predictor of the use of modern contraceptive for FP practices. Therefore, it can be hypothesized that:

**Hypothesis** **1** **(H1).** *Contraceptive self-efficacy has a positive impact on the adoption of modern contraceptive methods for FP practices*.

### 2.2. Contraceptive Knowledge and Family Planning (FP) Practices

Contraceptive knowledge was defined by Nsubuga et al. [36] as “the state of awareness of contraceptive methods, any specific types and the source of contraceptive”. Contraceptive knowledge enables women to easily access FP services [37]. It is reported that counselling increases contraceptive awareness, which modifies people’s attitudes towards the use of contraceptives [38]. Efficient contraceptive knowledge helps in changing people’s perceptions and decisions about FP [39]. Researchers have also found that educated women are more aware of contraceptive methods and FP practices, which ultimately increases the use of contraceptives among females [40]. It is also reported that females with good contraceptive knowledge practiced different methods effectively [41]. In contrast, individuals with a lack of contraceptive knowledge will discontinue contraceptive use due to its side effects or method failures [42]. According to a recent survey, 3/4th of the overall urban population is aware of FP practices, but a low level of awareness among rural population was reported [16]. Well-aware and knowledgeable individuals regarding different contraceptive methods have a tendency to solve different FP issues [43,44,45], such as intercourse and the method not changing the woman’s menstrual periods [46], intrauterine device and implant [47], and female sterilization [48].

Contraceptive knowledge in terms of awareness about the available contraceptive methods helps people in choosing the best and effective contraceptives practices, and also changes people’s fertility preferences [49]. It has been noted that people who are aware of implants and breastfeeding as contraceptive methods were more interested in the adoption of modern contraceptive methods for FP practices [50]. Studies in the context of a developing country, such as Pakistan, highlighted the gap between contraceptive knowledge and FP practice [17,51]. This gap is because of a lack of knowledge about the benefits and availability, as well as misinformation, of modern contraceptive methods for FP practices. Major sources delivering contraceptive knowledge include healthcare centers, friends, family, and media [52]. Therefore, based on the available literature, it can be hypothesized that:

**Hypothesis** **2** **(H2).** *Contraceptive knowledge has a positive impact on the adoption of modern contraceptive methods for FP practices.*

### 2.3. Spousal Communication and Family Planning (FP) Practices

Backman, as cited in [53], stated that “spousal communication in the marital dyad is generally defined as the frequency of discussion between spouses, as reported by one or both partners” (p. 5). Communication between spouses plays an important role in the continuous adoption of modern contraceptive methods for FP practices. Partner communication appeared as a topic of interest regarding FP practices. In this regard, researchers found a positive association between spousal communication and FP practices [54,55,56]. Another study reported husbands as key decision makers for getting access to health and FP services. A husband’s education level is significantly associated with the current use of contraceptives. The location of service providers, the quality of services, women’s age, and financial status also determine the use of contraceptives [4].

FALAH (Family Advancement for Life and Health) is already working on male involvement in FP programs. An analysis of program outcomes found that engaging Pakistani men in FP practices to support and encourage their wives to use FP services and introducing male contraceptive methods can increase the utilization and acceptance of FP practices among the population [57]. Similarly, Khan et al. [58] stated that husband approval is a strong predictor of the use of contraceptives. Spousal communication helps in coping with psychological barriers and reduces emotional strains that discourage the use of contraceptives [59]. It helps couples in decision making concerning an appropriate family size, and enhances positive intentions towards modern contraceptive methods for FP practices. Thus, it can be hypothesized that:

**Hypothesis** **3** **(H3).** *Spousal communication has a positive impact on the adoption of modern contraceptive methods for FP practices.*

### 2.4. Moderating Role of Perceived Barriers

Glasgow [60] defined perceived barriers as “A person’s estimation of the level of challenge of social, personal, environmental, and economic obstacles to a specified behavior” (p. 1). In the literature, the concept of perceived barriers has been extensively used with the health belief model (HBM). Perceived barriers have been used in many theories, including HBM, social cognitive theory, and social-ecological theory [60]. The integrated impact of multiple barriers hamper women from accessing reproductive health services. The restricted mobility of women by family [42] and a lack of communication between couples are factors that hamper women from using contraceptives [61]. Additionally, barriers restrain women’s ability to practice contraceptive methods. Most of the time, women that desire to limit their fertility by using contraceptives are influenced by religious and cultural hindrances [11,62]. They have to face great resistance from social barriers comparative to financial issues [63,64].

Women’s perceptions about contraceptive use, fear of their husbands’ negative response, and FP practices are perceived as an unacceptable act by society; therefore, culture limits the use of contraceptives among women [65]. Another study conducted by researchers in Pakistan declared that reasons for not using contraceptives include a desire for a baby boy (19%), fear of a health risk (29%), and lack of partner support and consideration of them as un-Islamic (14%); similar findings were found in other studies [66,67]. Interpersonal violence [68], cost, shyness, desire for a baby boy and a large family size [69], fear of sin, sterility [70], misinterpretation, husband and in-laws disapproval, prevailing myths, and social norms are all factors that contribute to the low intention of adopting of FP practices [66,71].

Fear of privacy breach, stigmatization, and FP service providers’ attitudes negatively affect the adoption of modern FP practices among women, despite them having knowledge about contraceptive use [72,73]. Spousal communication increases FP practices, but in-laws’ pressure, low parity, and administrative issues weaken this relationship [74]. Men’s disinterest and lack of knowledge about contraceptives, female financially dependency, and physical violence discourage women to communicate with their husbands about FP practices, which ultimately causes the low prevalence or lack of use of contraceptive methods [75]. Despite having information about several available FP methods, a low use of contraceptives has been noted among couples of rural areas due to misconceptions about risks associated with contraceptive methods [76]. Family environments also define women’s behavior towards FP practices [77]. A woman’s autonomy to make decisions about any aspect of her life is strongly influenced by the stratified family structure [78]. All these barriers contribute towards modern contraceptive prevalence for FP practices, in which women do not want to conceive for a period of time but still do not use any contraceptives [79]. Based on the above literature, it is argued whether perceived barriers act as moderator in the relationship between CSE, contraceptive knowledge, spousal communication, and FP practices or not. Therefore, it can be hypothesized that:

**Hypothesis** **4** **(H4).** *Perceived barriers moderate the relationship between contraceptive self-efficacy and the adoption of modern contraceptive methods for FP practices.*

**Hypothesis** **5** **(H5).** *Perceived barriers moderate the relationship between contraceptive knowledge and the adoption of modern contraceptive methods for FP practices.*

**Hypothesis** **6** **(H6).** *Perceived barriers moderate the relationship between spousal communication and the adoption of modern contraceptive methods for FP practices*.

**Figure 1:**
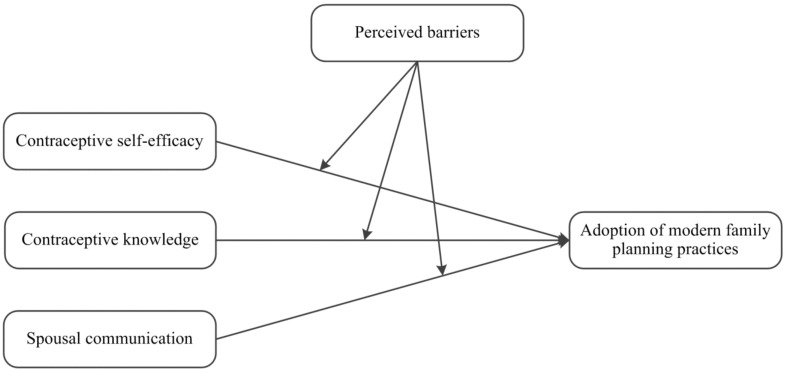
Research model.

The research model of the study is presented in Figure 1.

## 3. Methodology

### 3.1. Sample and Data Collection

Women of reproductive age are the main target of FP practices in Pakistan due to higher needs for the use of contraceptives at this age. The adoption of modern contraceptive methods for FP is a key variable in current research. Using a convenience sampling technique, data were collected from married women of reproductive age from the Rawalpindi and Neelum Valley regions in Pakistan through distributed questionnaires. Convenience sampling has the advantages of being inexpensive, efficient, and easy to use. We selected the aforementioned sampling locations because both these regions are highly prevalent in terms of FP practices. Additionally, the travel restrictions implemented during the COVID-19 outbreak made it difficult for the authors to visit other areas for data collection. We decided to collect data using both self-administered questionnaires and social circles from these areas to distribute our questionnaire to the relevant samples. A cover letter was attached, declaring the purpose of the research and asking participants at the time they join the study for relevant and historical information on spousal communication and decision making regarding FP practices. A screening question was also placed at the beginning of the survey to clearly ask whether respondents belonged to these regions and they knew the contraceptive methods used in FP practices. Confidentiality, anonymity, and voluntary participation were also ensured.

A total number of 340 questionnaires were distributed. The authors believe that the sampling size was appropriate due to the COVID-19 restrictions and respondents’ hesitation to respond to specific questions because of cultural and religious beliefs [11]. Out of the 292 questionnaires that were returned 42 were not useable, making the valid response rate 73.5%. The contraceptive prevalence rate in our sample was 41.28%.

As shown in Table 1, the majority of the women participants were literate (86.8%), most were non-working (63.6%), the majority of the women were in the age range of 24 to 35 years (78.3%), and the majority of the women got married in the age range of 18–25 years (72%). Most of the participants were residents of a rural area (70%), and most were Muslim (95.6%). The majority of the participants’ husbands were literate (95.2%) and working (97.2%). Of the respondents, 48% of them had a maximum of two–three children and (25%) had four or more children. Of the women, 92% of them reported having a good health status and 72.4% reported that their husbands were the head of the household. Of the respondents, 62.3% responded that their husbands were highly involvement in decision making regarding pregnancy, while 64.8% responded that they have spousal communication regarding FP and birth spacing.

### 3.2. Measurements

All the study variables were measured on a 5-point Likert scale. All constructs were measured on a Likert scale ranging from strongly disagree = 1 to strongly agree = 5.

Constructs such as contraceptive self-efficacy (CSE) were measured using a 7-item scale developed by Prata et al. [80]. One sample item which was measured was “I can use a modern contraceptive method to prevent pregnancy”. Contraceptive knowledge (CK) was measured by using a 7-item scale developed by Lincoln et al. [81]. One sample item which was measured was “I am aware that health education is important for women who want to use contraception”. Spousal communication (SC) was measured using a 5-item scale developed by Wegs et al. [82]. One sample item which was measured was “I and my spouse discuss things that happened during the day”. Modern FP practices were measured using a 7-item scale developed by Lincoln, Mohammadnezhad, and Khan [81]. One sample item which was measured was “I often use one of the contraceptives to prevent unplanned pregnancy”. Perceived barriers (PB) were measured using a 14-item scale developed by Sen et al. [83]. One sample item which was measured was “Contraceptive measures are too expensive for me”. The details of all constructs and their corresponding items are presented in Appendix A, Table A1. According to the criteria defined by Fornell and Larcker [84], the composite reliability values for all constructs were above the threshold (i.e., 0.70).

### 3.3. Common Method Bias

A common bias test was performed by taking into account Harman’s single factor [85]. Five constructs with their corresponding non-removed items were tested using an exploratory factor analysis by Harman’s single-factor test and analyzed with an unrotated factor solution. It was shown that there is no question about the common method bias in the current research data due to no emerging factor being reported, and 41.451% (less than 50%) variance was documented for the first factor, as suggested by Podsakoff, MacKenzie, Lee, and Podsakoff [85].

### 3.4. Control Variables

A one-way ANOVA was performed to control the variation in the adoption of modern contraceptive methods for FP practices on the basis of demographic variables used in the study. Results obtained from one-way ANOVA (see Table 2) indicated no significant differences in the adoption of contraceptive methods for FP practices (dependent variable) across qualification (F = 0.880, *p* > 0.05), profession (F = 3.371, *p* > 0.05), age at time of marriage (F = 2.881, *p* > 0.05), religion (F = 1.495, *p* > 0.05), health status (F = 1.267, *p* > 0.05), husband’s qualification (F = 1.496, *p* > 0.05), husband’s profession (F = 0.897, *p* > 0.05), and head of household (F = 0.399, *p* > 0.05).

At the same time, the one-way ANOVA indicated significant differences in FP across region (F = 19.089, *p* < 0.05), area of residence (F = 19.089, *p* < 0.05), current age (F = 2.682, *p* < 0.05), and number of children (F = 7.984, *p* < 0.05). Subsequently, factors identified as significant were entered as control variables in step 1 of a regression analysis for a single dependent variable.

## 4. Results

Means, standard deviations, scale reliabilities (**bold diagonal entries**)**,** and correlation matrices are presented in Table 3. Reliabilities for all constructs were greater than the cutoff value (i.e., α ≥ 0.7), which indicates acceptable reliability [86]. The results also revealed that all the absolute values of the correlation coefficients and the VIF statistics for each individual variable are less than 0.5 and 10, respectively [86]. Hence, multicollinearity is not a serious problem in the study, and the results are reliable. Table 3 also indicates that CSE is significantly positively correlated with modern FP practices (r = 0.48, *p* < 0.01) providing support for proposed hypothesis 1. Contraceptive knowledge is significantly positively correlated with modern FP practices (r = 0.34, *p* < 0.01), which provides support for proposed hypothesis 2. Modern FP practices are significantly positively correlated with spousal communication (r = 0.22, *p* < 0.01), which provides support for proposed hypothesis 3. Perceived barriers are not correlated with modern FP practices (r = 0.092, *p* = ns). Control variables, such as area of residence, region, current age, and number of children are positively correlated with modern FP practices.

A multiple regression analysis was run to check the relationship between variables in the proposed model of this study. Table 4 shows the results of the regression analysis for the controls, direct effects, and moderating variable. The findings reveal that control variables, such as area of residence (β = 0.126, *p* < 0.01), region (β = 0.256, *p* < 0.05), current age (β = 0.325, *p* < 0.01), and number of children (β = 0.258, *p* < 0.05) significantly influence modern FP practices. The results show a significant positive impact of CSE on the adoption of modern contraceptive methods for FP practices (β = 0.551, *p* < 0.001). Thus, hypothesis 1 is accepted. The regression analysis shows that there is a significant positive impact of contraceptive knowledge on the adoption of modern contraceptive methods for FP practices as (β = 0.226, *p* < 0.01); thus, hypothesis 2 is accepted. In addition, the results indicate that spousal communication has a significant positive impact on the adoption of modern contraceptive methods for FP practices as (β = 0.184, *p* < 0.01), thus leading towards the acceptance of hypothesis 3. Analysis shows that perceived barriers have no significant direct effect on the adoption of modern contraceptive methods for FP practices as (β = 0.049, *p* = ns).

Hypotheses 4, 5, and 6 were tested using moderated regression analysis. Where control variables were entered in step 1, independent and moderator variables were entered in step 2, and interaction terms were entered in step 3. Results show that in the third step after incorporating for interaction terms, such as contraceptive self-efficacy×perceived barriers, the results (β = 0.168, *p* < 0.05) lead to the rejection of hypothesis 4, that higher perceived barriers weaken the relationship between contraceptive self-efficacy and the adoption of modern contraceptive methods for FP practices in such a way that the relationship is weaker when the perceived barrier is high.

Result shows that FP practices in women with high CSE will be higher even in the presence of high perceived barriers. In addition, regression analysis shows that by incorporating interaction terms in the model for contraceptive knowledge×perceived barriers (β = −0.020, *p* = ns) and for spousal communication×perceived barriers (β = 0.037, *p =* ns) in the model, hypotheses 5 and 6 are not accepted. These results indicate that perceived barriers are not moderating the relationship between contraceptive knowledge and the adoption of modern contraceptive methods for FP practices or that between spousal communication and the adoption of modern contraceptive methods for FP practices.

**Figure 2:**
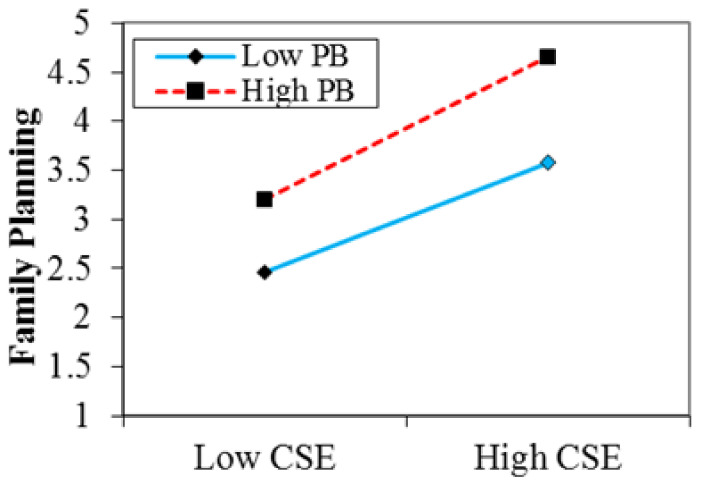
Interactive effect of contraceptive self-efficacy and perceived barriers on FP practices. CSE = contraceptive self-efficacy; PB = perceived barriers.

The interaction effect in Figure 2 shows that the relationship between CSE and the adoption of modern FP practices was stronger in the presence of high perceived barriers (in dashed red line) than in the presence of low perceived barriers (in solid blue line); thus, hypothesis 4 is rejected.

## 5. Discussion 

The purpose of this study was to investigate the causal effect of different factors (i.e., CSE, contraceptive knowledge, and spousal communication) that influence the adoption of modern contraceptive methods for FP practices. Additionally, the moderating role of perceived barriers was also examined in the relationships between aforementioned constructs [31,32]. The findings were in support of previous studies conducted by scholars [20,25], where similar findings were reported.

Contraceptive knowledge as awareness was found to have a significant positive impact on the adoption of modern contraceptive methods for FP practices. These findings were in line with previous studies findings [37,40]. This is because contraceptive knowledge among women encourages them to adopt modern methods for FP services and choose suitable method for practice. A good level of contraceptive knowledge improves the modern contraceptive prevalence. Contraceptive knowledge modifies people’s perceptions about FP practices [39]. Furthermore, the majority respondents were literate, so they valued contraceptive knowledge as an important factor for FP practices. Thus, it is quite logical to infer that the adoption of modern contraceptive methods for FP in Pakistan can be enhanced by increasing comprehensive knowledge about contraceptive measures among women.

Similarly, spousal communication also has a positive impact on the adoption of modern contraceptive methods for FP practices. Spousal communication is an effective way to involve males in FP practices and support women’s decisions about fertility preferences. Partner support and encouragement is a key determinant of FP practices [87]. The current findings were in line with previous studies [54,55,56,88]. As discussed in the literature, good spousal communication and encouragement by their partners allows women to make decisions about desired family size, usability, selection, and awareness of all available FP methods, which results in a reduction in contraceptive discontinuation and their low prevalence. This situation usually happens because of public dissatisfaction and a fear of opposition. Introducing male-oriented FP methods could help in increasing the uptake of FP practices by couples.

The results of moderated regression analysis show that the relationship between CSE and the adoption of modern contraceptive methods for FP practices is moderated by perceived barriers. Since the perceived barriers were used as moderators between the relationship of CSE and modern FP practices for the first time, the findings of the current study are supported by evidence from previous studies [20,25,61], where they declared that women with higher CSE are motivated and can convince men to use contraceptives. The adoption of any health behavior is dependent on individuals’ intentions to adopt that specific behavior. If an individual has strong intentions to practice or adopt a specific health behavior as well as the self-efficacy to overcome his/her perceived obstacles, the probability to adopt a specific health behavior increases [89,90]. As in the current study, participants reported higher CSE; therefore, the presence of barriers cannot reduce their intentions to practice modern FP methods.

The results of the interactive effect of perceived barriers and contraceptive knowledge show that perceived barriers do not moderate the relationship between contraceptive knowledge and the adoption of modern FP practices, which contradicts a proposed hypothesis. This result is in accordance with the common-sense model [91]. The model explains that human behavior is determined by the process of learning. Before adopting any health behavior, an individual assesses its pros and cons through cognition. For example, if individuals have to get treatment for a disease they will think about its cost, prognosis, and benefits, and then make decisions about action. Comprehensive knowledge about threats associated with health behavior reduces fear and leads to the adoption of that behavior [92]. As the participants of this study reported a higher level of contraceptive knowledge, it can thus be concluded, based on the previous literature, that high contraceptive knowledge among women helps them to make informed choices, overcome fears, and motivate them towards adopting modern FP practices.

The results of the interactive effect between perceived barriers and spousal communication were not significant, which shows that perceived barriers were not moderating the relationship between spousal communication and the adoption of modern FP practices. Since the literature shows that spousal communication about using contraceptives and involving the male partner in decision making about fertility preferences directly influences efforts for limiting fertility, they help women in overcoming perceived barriers as the fear of opposition is being shared by both partners [93]. Evidence from previous studies [94,95] also reveals that dynamics of spousal communication have a positive effect on contraceptive behavior; thus, these result are in line with the findings of the current study. Spousal discussion boosts modern FP use and consequently reduces fertility and maternal mortality rate.

### 5.1. Practical Implications

The findings provide several implications for practice. It is recommended that policymakers should incorporate modern contraceptive FP program models as a strategy to enhance the contraceptive prevalence rate. Special consideration should be given to spousal communication, and couples should be encouraged to discuss the adoption of modern contraceptive methods for FP practices. Awareness campaigns should be launched that highlight the benefits of spousal discussion about ideal family size, societal pressures, complications related to closely spaced deliveries, unsafe abortion, the risks of maternal and child mortality, malnutrition among children, and modern FP practices. Policymakers should also formulate policies for male involvement in modern FP programs across the country by introducing improved male-oriented methods and incorporating male FP workers to reduce communication barriers and shyness (as shown by a program that has been launched by FALAH in Pakistan and reported positive outcomes) [57]. FP program stakeholders should focus on promoting contraceptive knowledge among women to promote the adoption of modern contraceptive methods for FP practices.

Understanding different factors in the adoption of modern FP practices is necessary in formulating more suitable policies for public health [8,96]. As the use of FP is high in educated and urbanized people, there is a need to focus on slums and rural areas with a low literacy rate as well as how their perceptions about ideal family size change [88]. As the findings indicated that improving contraceptive knowledge leads towards FP practices, this study provides baseline information to policymakers towards the value of gaining comprehensive knowledge to increase the use of FP [97]. This study also draws public attention towards spousal discussion because it can increase the usage of modern methods for FP among couples, leading to a reduction in unwanted pregnancies and associated risks. In addition, our findings highlight the need for proper fund allocation as well as the provision of training and refresher courses for female health workers [98]. Furthermore, counselling intervention should be introduced to involve in-laws in programs to reduce barriers toward the adoption of modern methods for FP practices [99,100]. This study attempts to assist the Pakistani government in reaching its national development goals of enhancing maternal and reproductive health through the increased use of modern contraceptives.

### 5.2. Limitations and Directions for Future Research

This paper has several limitations. First, the findings of current study were predisposing to recall bias as data were self-reported by respondents rather than dyads, etc. Future studies should ensure that the way questions are worded does not influence the answers of participants due to the possible risk of recall bias. Second, as the majority of the respondents belonged to the Rawalpindi and Neelum Valley regions, the findings may not be generalizable due to the smaller sample size and convenience sampling technique using a specific targeted group, which lack external validity. Future studies should run the analysis using a larger dataset. Third, the current study is limited and not able to measure several other important variables (i.e., the desire for an additional child, myths/misconceptions, fear of side effects, and partner/friend discouragement) which also affect the use of contraceptives. Future researchers are required to conduct studies on the approval of modern FP practices by couples and their association with contraceptive knowledge and barriers in acquiring contraceptive knowledge. Fourth, since the current study employed a statistical method due to the authors’ limitations in using advanced statistical tools, future studies may use PLS-SEM as an advanced statistical tool, which seems much more appropriate, especially when analyzing possible moderation. Finally, for formulating comprehensive strategies about couple counselling to overcome the knowledge and practice gap and to dispel misconceptions about contraceptives, researchers should conduct qualitative studies on spousal communication and contraceptive knowledge.

## 6. Conclusions

To conclude, the empirical analysis supported three hypotheses proposed in this study. The results indicated that CSE, contraceptive knowledge, and spousal communication positively impact the adoption of modern contraceptive methods for FP practices. In particular, the higher CSE in women motivates them to adopt modern contraceptive methods for FP practices. It also encourages women to overcome all the barriers, which limit their access to FP services. CSE helps women to understand the importance of FP practices that are important in maintaining the gap between child births. It supports women in decision making about fertility preferences, which helps them to recover their health from previous pregnancies and provide better care to their children. 

## Figures and Tables

**Table 1:** Socio-demographic characteristics of respondents.

Characteristics	N (250)	*n* (%)
**Women’s Education**		
Illiterate	33	13.2%
Literate	217	86.8%
**Employment Status** (**Wife**)		
Employed women	91	36.4%
Unemployed women	159	63.6%
**Age of Women** (**Years**)		
≤24	37	14.6%
>24 to 35	195	78.3%
>35	18	7.1%
**Age of Women at Time of Marriage**		
>25	67	26.9%
>18 to 25	180	72%
≤18	3	1.1%
**Area Demographics**		
Urban areas	75	30%
Rural areas	175	70%
Religion		
Muslim	239	95.6%
Non-Muslim	11	4.4%
**Husbands’ Education**		
Illiterate	238	95.2%
Literate	12	4.8%
**Employment Status** (**Husband**)		
Employed husband	243	97.2%
Unemployed husband	7	2.8%
**Number of Living Children**		
0–1 child	68	27%
2–3 children	120	48%
4 or more children	62	25%
**Health Status**		
Healthy	230	92%
Unhealthy	20	8%
**Household Head**		
Husband	181	72.4%
Wife	69	27.6%
**Decision Making Regarding Pregnancy**		
Husband decides	156	62.3%
Mother-in-law decides	4	1.6%
Respondent (woman) decides	21	8.5%
Both (husband and wife) decide	69	27.6%
**Spousal Communication Regarding Family Planning and Birth Spacing**		
No	88	35.2%
Yes	162	64.8%

**Table 2:** One-way ANOVA.

	Modern Family Planning Practices	
Source of Variation	F-Statistic	*p*-Value
Qualification	0.880	0.510
Profession	3.371	0.068
Area of residence	19.089	0.000
Region	19.089	0.000
Current age	2.682	0.047
Age at time of marriage	2.881	0.091
Religion	1.495	0.226
Husband’s qualification	1.496	0.180
Husband’s profession	0.897	0.354
No. of children	7.984	0.000
Health status	1.267	0.261
Head of household	0.399	0.754

**Table 3:** Means, standard deviations, correlations, and reliabilities.

Variables	1	2	3	4	5	6	7	8	9	10	11	12	13	14	15	16	17
1. CSE	(0.83)																
2. CK	0.413 **	(0.80)															
3. SC	0.129 *	0.321 **	(0.78)														
4. FP	0.481 **	0.344 **	0.223 **	(0.97)													
5. PB	0.006 ns	0.236 **	0.106 ns	0.092 ns	(0.75)												
6. Qual.	0.041 ns	0.012 ns	0.023 ns	0.025 ns	0.037 ns	1.00											
7. Prof.	0.231 ns	0.125 ns	0.145 ns	0.236 ns	0.061 ns	0.652 ns	1.00										
8. AoR	0.062 **	0.054 *	0.031 *	0.027 **	0.014 **	0.031 *	0.045 **	1.00									
9. Reg.	0.265 **	0.222 **	0.256 *	0.362 **	0.451 *	0.325 **	0.322 *	0.316 **	1.00								
10. CA	0.126 *	0.215 *	0.279 *	0.043 **	0.201 *	0.006 *	0.325 *	0.122 **	0.421 **	1.00							
11. ATM	0.011 ns	0.022 ns	0.043 ns	0.054 ns	0.134 ns	0.147 ns	0.242 ns	0.327 ns	0.362 ns	0.370 ns	1.00						
12. Relig.	0.12 ns	0.42 ns	0.20 ns	0.07 ns	0.33 ns	0.013 ns	0.52 ns	0.103 ns	0.321 ns	0.254 ns	0.115 ns	1.00					
13. HQ	0.33 ns	0.11 ns	0.256 ns	0.125 ns	0.269 ns	0.112 ns	0.325 ns	0.225 ns	0.124 ns	0.254 ns	0.365 ns	0.105 ns	1.00				
14. HP	0.269 ns	0.171 ns	0.002 ns	0.185 ns	0.125 ns	0.145 ns	0.062 ns	0.069 ns	0.065 ns	0.025 ns	0.032 ns	0.277 ns	0.253 ns	1.00			
15. NC	0.107 **	0.116 *	0.223 *	0.178 *	0.121 *	0.452 **	0.128 *	0.248 **	0.179 **	0.125 *	0.326 *	0.028 **	0.369 **	0.459 **	1.00		
16. HS	0.025 ns	0.036 ns	0.269 *	0.002 ns	0.003 ns	0.003 ns	0.045 ns	0.010 ns	0.019 ns	0.018 ns	0.017 ns	0.369 ns	0.269 ns	0.369 ns	0.269 ns	1.00	
17. HH	0.012 ns	0.009 ns	0.23 ns	0.051 ns	0.023 ns	0.021 ns	0.026 ns	0.027 ns	0.025 ns	0.034 ns	0.317 ns	0.212 ns	0.415 ns	0.025 ns	0.145 ns	0.259 ns	1.00
Mean	3.16	3.59	3.31	3.14	2.15	2.87	1.98	1.22	2.58	2.67	2.35	0.567	2.50	2.89	3.00	0.61	0.67
S.D	0.69	0.59	0.99	0.87	0.82	0.78	0.61	0.69	0.23	0.25	0.49	0.06	0.71	0.55	0.96	0.03	0.11

Notes: *n* = 250; alpha reliabilities are given in parentheses. *p* < 0.05. S.D = standard deviation, CSE = contraceptive self-efficacy, CK = contraceptive knowledge, SC = spousal communication, PB = perceived barriers, Qual = qualification, Prof. = profession, AoR = area of residence, Reg. = region, CA = current age, ATM = age at time of marriage, Relig. = religion, HQ = husband’s qualification, HP = husband’s profession, NC = No. of children, HS = health status, and HH = head of household. **, correlation is significant at the 0.01 level; *, correlation is significant at the 0.05 level. ns = correlation is not significant.

**Table 4:** Hierarchical moderated regression analysis.

	**Modern Family Planning Practices**		
**Predictors**	**Β**	**R^2^**	**∆R^2^**
**Step 1**			
Control variables		0.082	
Qualification	0.065 ns		
Profession	0.01 ns		
Area of residence	0.126 **		
Region	0.256 *		
Current age	0.325 **		
Age at time of marriage	0.125 ns		
Religion	0.144 ns		
Husband’s qualification	0.136 ns		
Husband’s profession	0.225 ns		
No. of children	0.258 *		
Health status	0.452 ns		
Head of household	0.201ns		
**Step 2**			
Contraceptive self-efficacy	0.551 ***	0.448	0.366 ***
Contraceptive knowledge	0.226 *		
Spousal communication	0.184 **		
Perceived barriers	0.049ns		
**Step 3**			
CSE × PB	0.168 **	0.442	0.016 ns
CK × PB	−0.020 ns		
SC × PB	0.037 ns		

Notes: ***, *p* < 0.001; **, *p* < 0.01; and *, *p* < 0.05. CSE = contraceptive self-efficacy, CK = contraceptive knowledge, SC = spousal communication, and PB = perceived barriers. ns = not significant.

## Data Availability

The data used to support the findings of this study are available from the corresponding author upon request.

## Appendix A

**Table A1:** Constructs along with their corresponding items.

Construct and Items	Source
**Contraceptive Self-Efficacy** (**CSE**)	[80]
**CSE1:** I can use a modern contraceptive method to prevent pregnancy.	
**CSE2:** I can consistently use (method of interest).	
**CSE3:** I feel confident that I can obtain an effective birth spacing method.	
**CSE4:** I can talk to my partner about using modern contraceptive to prevent pregnancy.	
**CSE5:** I feel comfortable talking with a health care provider about birth space method.	
**CSE6:** I can convince my partner to use the modern FP practices.	
**CSE7:** I can use modern FP practices even if my partner disagrees.	
Contraceptive Knowledge (CK)	[81]
**CK1:** I use birth control pills that are effective even if I misses taking them for two or three days in a row.	
**CK2:** I believe female sterilization is one way to avoid pregnancy.	
**CK3:** I am aware that health education is important for women who want to use contraception.	
**CK4:** I believe the contraceptive pills do not guarantee 100% protection.	
**CK5:** If I feel the side effects of using one kind of contraceptive pill, I will be switching to another type that might help me.	
**CK6:** I believe using both a condom and the pill is a very effective contraceptive.	
**CK7:** I believe the pill increases a woman’s risk of ovarian, endometrial or cervical cancer.	
**Spousal Communication** (**SC**)	[82]
**SC1:** I and my spouse discuss things that happened during the day.	
**SC2:** I and my spouse often discuss worries or feelings.	
**SC3:** I and my spouse often discuss what to spend household money on.	
**SC4:** I and my spouse discuss when to have children.	
**SC5:** I and my spouse discuss whether to use modern FP practices or not.	
**Family Planning** (**FP**) **practices**	[81]
**FP1:** I often visit a health center for FP services.	
**FP2:** I often use one of the contraceptives (**A**) to prevent unplanned pregnancy.	
**FP3:** I had any unplanned pregnancy due to lack of contraceptive (**A**) use.	
**FP4:** I use contraceptives (**A**) every time when I do not intend to get pregnant.	
**FP5:** I use different types of contraceptives (**A**).	
**FP6:** My current method of contraceptives (**A**) changes from time to time.	
**FP7:** I often practice traditional contraceptive methods including herbal and breast feeding if I do not use any contraceptives (**A**).	
**Perceived Barriers** (**PB**)	[83]
**PB1:** Contraceptive (**A**) use is not suitable for me.	
**PB2:** Contraceptive use (**A**) may be painful for me.	
**PB3:** Contraceptive use (**A**) is time-consuming for me.	
**PB4:** Contraceptive use (**A**) disturbs my sex life.	
**PB5:** Contraceptive measures (**A**) are too expensive for me.	
**PB6:** I am concerned about having a bad reaction by using contraceptive measures (**A**).	
**PB7:** Prolonged use of contraceptive measures (**A**) affects me negatively.	
**PB8:** Contraceptive measures (**A**) affect my husband negatively.	
**PB9:** Contraceptive measures (**A**) affect attitudes of people towards me negatively.	
**PB10:** I find it embarrassing to use contraceptive measures (**A**).	
**PB11:** Contraceptive use (**A**) does not fit in with our culture.	
**PB12:** I believe the contraceptive use (**A**) is not hygienic.	
**PB13:** My husband does not want contraceptive use (**A**).	
**PB14:** I cannot talk to a male health professional about contraceptive use (**A**).	

Contraceptive Methods (A): Pill, IUCD, condom, periodic abstinence, withdrawal, female sterilization, male sterilization, implants.
